# Quality of Life among Persons with HIV/AIDS in Iran: Internal Reliability and Validity of an International Instrument and Associated Factors

**DOI:** 10.1155/2012/849406

**Published:** 2012-01-12

**Authors:** Pedram Razavi, Kaveh Hajifathalian, Behtash Saeidi, Gholamreza Esmaeeli Djavid, Mehrnaz Rasoulinejad, Mahboube Hajiabdolbaghi, Koosha Paydary, Parastoo Kheirandish, Mahsa Foroughi, SeyedAhmad SeyedAlinaghi, Minoo Mohraz, Willi McFarland

**Affiliations:** ^1^Department of Medicine, University of Southern California, CA, USA; ^2^Department of Preventive Medicine, University of Southern California, CA, USA; ^3^Iranian Research Center for HIV/AIDS (IRCHA), Tehran University of Medical Sciences, Tehran, Iran; ^4^Academic Center for Education, Culture and Research (ACECR), Tehran, Iran; ^5^CAPS and Institute for Global Health, University of California, San Francisco, CA 94105, USA

## Abstract

The purpose of this cross-sectional study on 191 HIV/AIDS patient was to prepare the first Persian translation of complete WHOQOL-HIV instrument, evaluate its reliability and validity, and apply it to determine quality of life and its associated factors in Tehran, Iran. Student's *t*-test was used to compare quality of life between groups. Mean Cronbach's **α** of facets in all six domains of instrument were more than 0.6 indicating good reliability. Item/total corrected correlations coefficients had a lower limit of more than 0.5 in all facets except for association between energy and fatigue facet and physical domain. Compared to younger participants, patients older than 35 years had significantly lower scores in overall quality of life (*P* = 0.003), social relationships (*P* = 0.021), and spirituality/religion/personal beliefs (*P* = 0.024). Unemployed patients had significantly lower scores in overall quality of life (*P* = 0.01), level of independence (*P* = 0.004), and environment (*P* = 0.001) compared to employed participants. This study demonstrated that the standard, complete WHOQOL-HIV 120 instrument translated into Farsi and evaluated among Iranian participants provides a reliable and valid basis for future research on quality of life for HIV and other patients in Iran.

## 1. Introduction

Quality of life is defined not only by one's general health but also by psychological well-being and social status. The psychosocial aspects of quality of life may be increasingly important in patients with HIV infection as the disease becomes more chronic in nature during the era of more effective antiretroviral treatment (ART). Studies show that HIV patients often experience a decline in quality of life due to factors other than disease stage and physical condition [1–4], such as poverty, addiction, depression, and violence [5]. Understanding such factors and their influence helps to establish better social services to address multidimensional issues related to quality of life in these patients. However, the measurement of quality of life with its diverse dimensions is complex. The World Health Organization disseminates a standardized quality-of-life instrument specifically adapted for HIV patients (WHOQOL-HIV) which has been widely used and shown to be a valuable tool for evaluating patients' perception of their quality of life [6].

According to the 2010 update of the UNAIDS/WHO epidemiological fact sheet [7], the estimated number of people living with HIV/AIDS in Iran is 80,000, and the figure appears to be increasing each year. Despite the magnitude of the HIV-induced health and social problems, little is known about quality of life and its determinants among Iranian patients living with HIV/AIDS. One study evaluated the quality of life in HIV/AIDS patients in Iran using an abbreviated WHO instrument (WHOQOL-BREF) [8]. To our knowledge, however, the complete WHOQOL-HIV instrument has not been yet translated to Farsi, assessed for its reliability, and applied to a patient population in Iran. Moreover, the influence of ART on quality of life has not been assessed in this population to date. Evaluation of the instrument in Iranian patients with HIV/AIDS and determining associated factors will help to better allocate limited available resources and address the social welfare needs of these patients.

In current study we sought to field test and assess the internal reliability and validity of complete WHOQOL-HIV instrument in Farsi and, if reliable, apply it to determine quality of life for patients with HIV in Tehran, Iran. In addition to demographic and social factors, we sought to examine any relationship between ART and quality of life in these patients.

## 2. Materials and Methods

This is a cross-sectional survey of patients living with HIV/AIDS visiting the Imam Khomeini Hospital consultation center for patients with behavioral disorders conducted over a one year period in 2009-2010. The purpose, procedures, and potential risks and benefits of the study were explained to patients, and all those agreeing to participate provided written informed consent. Parents provided written informed consent for minor participants. The study was approved by the hospital and the Tehran University Medical School ethics committee. The study was conducted in two stages. The first stage prepared a valid and reliable Farsi translation of the complete WHOQOL-HIV instrument, which was used to collect data during the second phase. The inclusion criteria were being 18 years of age or older, being an HIV-positive patient, and not having cognitive or communicative disabilities or psychotic disorders such as schizophrenia. Illiterate participants completed the form with assistance of an experienced clinician cooperating with the study.

The primary measure of this study is based on the standard quality-of-life questionnaire, the WHOQOL-HIV instrument, prepared and validated in field trials by the WHO for use in HIV populations. This instrument consists of 120 questions with response scales plus 38 importance items which are designed to assess patients' quality of life defined as “individuals' perceptions of their position in life in the context of the culture and value systems in which they live and in relation to their goals, expectations, standards, and concerns.” As shown in Table 1, the instrument consists of 6 domains and 29 facets and one general facet that measures overall quality of life and general health. Each facet consists of 4 individual items that are rated on a 5-point Likert's scale, where 1 indicates low and negative perceptions and 5 indicates high and positive perceptions. Facet scores are calculated as means of their item scores and range between 1 and 5. Domain scores are calculated as means of their facet scores multiplied by 4 and range between 4 and 20.

The questionnaire was translated to Farsi by two native speaking physicians with experience as medical literature translators. These were reviewed by two members of our research team to be easily understandable and were back translated independently to English by two other bilingual physicians. The prepared Farsi version was evaluated among 10 of the participants chosen at random followed by final revisions.

The reliability of the instrument was assessed using Cronbach's *α* for internal consistency. For structural validity, we calculated the corrected item's total correlation coefficients between the domains' total score and their facets' scores in addition to Pearson's correlation coefficients between the total scores of domains. For the analysis of correlates of quality of life, the following factors were evaluated as independent variables: gender, age, marital status (married, single, widowed, and divorced), level of education (illiterate, elementary, junior-high or high-school diplomas, and university degree), work status, risk factors for acquiring HIV (prison history, intravenous substance abuse, high-risk sexual behavior, and blood transfusion or donation), transmission routes (intravenous drug use, sex, vertical, and unknown), income, and ART use. Independent variables are reported as number and percent among participants. Facet and domain scores are presented as mean (± SD), and the student's *t*-test was used to compare means. Kolmogorov-smirnov's test and kurtosis/skewness were used to evaluate normal distribution of mean of scores. A *P*-value of less than 0.05 was considered significant. Data analysis was performed using SPSS (version 13.0; SPSS, Inc., Chicago, IL, USA).

## 3. Results

A total of 191 patients with HIV/AIDS were enrolled. Of these, 159 (83.2%) were male. The mean age was 35.7 years (±8.05) with a range of 11 to 60 years. Mean age of male participants did not differ from that of females (36.1 ± 8.2 versus 34.1 ± 7.1, respectively; *P* = 0.189). A summary of patients' characteristics is presented in Table 2. Of all participants, 95 (49.7%) were single and 69 (36.1%) married. The majority of patients report their level of education as having junior-high or high-school diplomas. From 191 patients, 113 (59.2%) had a history of incarceration, and 108 (56.5%) had history of intravenous substance abuse. The most common transmission modes were intravenous substance abuse (52.4%) and extramarital sexual relationship (25.1%). Of 191 participants, 75 (39.3%) were under highly active antiretroviral therapy (HAART). The mean number of family members of participants was 3.5 (±1.9), and 97 (50.8%) of them were heads of their households. Most (63.3%) patients declared that they have no income, and only 19 (10%) had monthly income of more than 200 dollars. Nearly half (44.5%) were home owners, 72 (37.7%) lived in rented houses, and 20 (10.5%) lived with their relatives.

As shown in Table 3, mean Cronbach's *α* of facets in all six domains were more than 0.6 indicating good reliability. The spirituality domain with Cronbach's *α* of 0.813 showed the highest internal consistency, whereas the physical domain with the Cronbach's *α* of 0.616 had the worst result. Item/total corrected correlations for association between each domain and their facets had a lower limit of more than 0.5 in all facets except for association between energy and fatigue facet and physical domain. Pearson's correlation coefficients for association between all domains, including overall quality of life and general health, were more than 0.4 in all instances.

Patients older than 35 years had significantly lower scores in overall quality of life (*P* = 0.003), social relationships (*P* = 0.021), and spirituality/religion/personal beliefs (*P* = 0.024) when compared to their younger counterparts (Table 4). Marital status and level of education did not show any significant association with any domain of quality of life. Unemployed patients had significantly lower scores in overall quality of life (*P* = 0.01), level of independence (*P* = 0.004), and environment (*P* = 0.001) compared to employed participants. Participants with any level of monthly income showed better quality of life in respect to level of independence compared to patients without income (*P* = 0.009). Number of family members, being the head of household, history of incarceration, or intravenous substance abuse, did not show any significant relation with different domains of quality of life (all *P* > 0.05). Quality of life did not differ significantly between patients using ART and untreated patients (*P* > 0.05).

## 4. Discussion

The present study was the first to assess the quality of life in a substantial number of patients living with HIV/AIDS in Iran using a field-tested Farsi version of the standard, complete WHOQOL-HIV 120 instrument. In the event, our Farsi version of the WHOQOL-HIV developed for this study had good reliability and validity. All main domains of questionnaire had Cronbach's *α* scores of more than 0.6, supporting an acceptable internal consistency for instrument. Structural validity assessed using item/total corrected correlations for association between each domain and their facets were in the range of 0.50–0.91 in all 29 facets with the exception of the energy and fatigue facet of the physical domain, and the correlations between individual domains of instrument were satisfactory with coefficients of more than 0.4.

In our study, employment status showed significant positive correlations with level of independence, spirituality/religion/personal beliefs, overall quality of life, and general health score among participants. Moreover, participants with monthly income reported a better level of independence compared to patients without financial sources in our study. These results underscore the importance of returning HIV/AIDS patients to employment as a significant step in improving their quality of life. In the era of ART, this return should be planned as a long-term one. As suggested by a previous study [4], working may provide a context for social support, identity, and meaning, accounting for observed better quality of life of employed subjects in our study. Indeed, multiple studies worldwide support our findings in Iran [9, 10]. However, the current economic environment presents many challenges to full employment in Iran.

The majority of patients in our study had history of imprisonment and/or intravenous substance abuse, with the latter being the route of transmission in the majority of participants as expected by the epidemiology of HIV in Iran [11, 12]. Intravenous drug users (IDUs) have multiple issues that have a bearing on the quality of life apart from HIV/AIDS that need to be considered in interpreting our results. These include simultaneously experiences such as addiction, poverty, depression, and high stigmatization [13]. Although previous studies have reported a negative relation between being an IDU and quality of life in HIV/AIDS patients [14, 15], in our study, we could not show any significant association between intravenous substance abuse and quality of life. This is in line with findings of Astoro et al., who did not find an association between being IDU and quality of life in HIV-positive patients [16]. The reason for this discrepancy regarding effect of intravenous drug abuse is not clear but could be due to different social profiles of IDUs in various cultures or our inability to discriminate between current and past users in this study.

We found that HIV/AIDS patients older than 35 years reported lower quality of life in the domains of social relationships and spirituality/religion/personal beliefs, as well as overall quality of life and general health. Older age has been shown to be associated with dissatisfaction with one's social relationships [17], and in a previous study Kalichman et al. suggested that HIV/AIDS patients who are older than 45 years experience significant emotional distress and thoughts of suicide [18]. Spirituality and social support may influence survival in patients with chronic disease [19]. Our result can be used to tailor supportive measures to match specific needs of an older subgroup of patients living with HIV/AIDS. Contrary to various studies which have shown better quality of life among HIV patients treated with antiretroviral drugs [20–22], this study did not show such an association. Some recent studies have suggested that ART may not affect health-related quality of life early in the course of therapy [6, 23, 24]. This, as well as relative small number of patients having AIDS and being on ART (17.1%), may be the cause of the observed results in our study.

The design of the present study does not allow us to deduce causality or determine the direction of the observed associations. Furthermore, the degree to which our results are representative of the whole population of Iranian HIV/AIDS patients is limited by the fact that our participants have been selected from a hospital-based consultation center in Tehran. In fact, convenient sampling could have hampered data generalization and, indeed, further limit applications of this instrument in a community-based level. For example, the fact that about 40% of patients were receiving HAART at the time study was conducted could have altered certain results and act as a possible source of bias. These apparent differences due to convenient sampling further increase the level of uncertainty in concluding whether generalization of our results to PLWHA is even plausible. Relative small number of female participants and patients with AIDS in this study should be considered before attributing results of this study to these populations.

To conclude, our study demonstrated that the standard, complete WHOQOL-HIV 120 instrument translated into Farsi and evaluated among Iranian participants provides a reliable and valid basis for future research on quality of life for HIV and other patients in Iran. Furthermore, the present study a significant positive relation between employment and several areas of quality of life and negative relation to older age. Such findings are examples that provide necessary information to take measures either by governmental or nongovernmental institutions to improve patients' quality of life. Although the causal relationship between these factors and the means to intervene need to be confirmed through prospective studies and intervention trials, we believe our study provides an important tool to make progress on improving the quality of life for persons with HIV/AIDS in Iran.

## Figures and Tables

**Table 1:** Quality-of-life measures including domains and facets, from the WHOQOL-HIV.

Domain I (physical)	Pain and discomfort
	Energy and fatigue
	Sleep and rest
	Symptoms of PLWHA*

Domain II (psychological)	Positive feelings
	Thinking, learning, memory, and concentration
	Self-esteem
	Bodily image and appearance
	Negative feelings

Domain III (level of independence)	Mobility
	Activities of daily living
	Dependence on medication or treatments
	Work capacity

Domain IV (social relationships)	Personal relationships
	Social support
	Sexual activity
	**Social inclusion**

Domain V (environment)	Physical safety and security
	Home environment
	Financial resources
	Health and social care: accessibility and quality
	Opportunities for acquiring new information and skills
	Participation in and opportunities for recreation/leisure activities
	Physical environment (pollution/noise/traffic/climate)
	Transport

Domain VI (spirituality/religion/personal beliefs)	SRPB
	**Forgiveness and blame**
	**Concerns about the future**
	**Death and dying**

*Facets that are highlighted in bold are specific to the persons living with HIV/AIDS and as such have been added to the original WHOQOL instrument.

**Table 2:** Participant characteristics, HIV/AIDS patients assessed for quality of life, Tehran, Iran, 2009-2010.

		Number	Percent
Gender	Male	156	83.2
	Female	35	16.8

Age	11–17	2	1.1
	18–24	8	4.2
	25–34	83	43.5
	35–44	69	36.1
	45 and older	29	15.1

Marriage	Single	95	49.7
	Married	69	36.1
	Divorced or widowed	27	14.2

Education	Illiterate	6	3.0
	Elementary	31	16
	Middle or high school	139	72.8
	University	15	8.2

Risk factors	Prison history	113	59.2
	Intravenous drug use	108	56.5
	Unprotected sex	53	27.7
	Blood transfusion and donation	32	16.7

Transmission*	Intravenous drug use	100	52.4
	Unprotected sex	48	25.1
	Vertical	1	0.5
	Unknown	19	9.9

HAART	Treated	75	39.3
	Untreated	116	60.7

Income	More than 200 dollars	19	10
	Less than 200 dollars	51	26.7
	No income	121	63.3

Employment	Employed	110	57.6
	Unemployed or retired	81	42.4

*The total percent is not 100 due to missing data.

**Table 3:** Domains mean Cronbach's *α* and correlation of their mean scores with participants' overall quality of life scores.

	Cronbach's *α*	Item/total correct correlation (min.–max.)	Correlation with overall quality of life
Domain I (physical)	*0.61*		0.522*
Pain and discomfort	**0.73**	**0.69–0.78**	
Energy and fatigue	**0.14**	−**0.05–0.78**	
Sleep and rest	**0.85**	**0.78–0.86**	
HIV symptoms	**0.74**	**0.57–0.84**	
Domain II (psychological)	*0.72*		0.652*
Positive feelings	**0.70**	**0.64–0.83**	
Cognition	**0.74**	**0.64–0.82**	
Self-esteem	**0.65**	**0.62–0.76**	
Body image and appearance	**0.70**	**0.64–0.81**	
Negative feelings	**0.80**	**0.63–0.91**	
Domain III (level of independence)	*0.78*		0.538*
Mobility	**0.66**	**0.70–0.76**	
Daily activities	**0.74**	**0.74–0.80**	
Medication and treatment dependency	**0.84**	**0.76–0.87**	
Aptitude for work	**0.89**	**0.83–0.90**	
Domain IV (social relationships)	*0.63*		0.705*
Personal relationships	**0.60**	**0.64–0.72**	
Social support	**0.67**	**0.68–0.74**	
Sexual activity	**0.66**	**0.61–0.84**	
Social inclusion	**0.59**	**0.61–0.74**	
Domain V (environment)	*0.65*		0.708*
Security	**0.47**	**0.50–0.73**	
Housing	**0.77**	**0.65–0.85**	
Financial resources	**0.74**	**0.69–0.84**	
Health and social work	**0.52**	**0.53–0.76**	
Learning opportunities	**0.67**	**0.65–0.79**	
Leisure opportunities	**0.81**	**0.76–0.81**	
Physical environment	**0.54**	**0.52–0.77**	
Transportation	**0.73**	**0.55–0.86**	
Domain VI (spirituality/religion/personal beliefs)	*0.81*		0.435*
Spirituality-religion and personal beliefs	**0.83**	**0.78–0.84**	
Forgiveness	**0.84**	**0.76–0.85**	
Spiritual connection and personal spiritual connection	**0.74**	**0.56–0.80**	
Death and dying	**0.84**	**0.77–0.85**	

*Pearson's *P* (2-tailed) <0.01.

**Table 4:** Domains' mean scores, compared between younger and older, employed and unemployed, and treated and naïve patients.

		Domain I	Domain II	Domain III	Domain IV	Domain V	Domain VI
Age	≥35	10.9 ± 2.1	11.9 ± 2.2	11.7 ± 2.7	10.7 ± 2.8^†^	10.2 ± 2.2	12.1 ± 3.1*
	<35	11.1 ± 2.3	12.3 ± 2.3	12.2 ± 2.1	11.6 ± 2.1^†^	10.8 ± 1.8	13.3 ± 3.3*
Employment	Employed	11.3 ± 1.9	12.2 ± 2.3	12.4 ± 2.3^♦^	11.4 ± 2.6	11.1 ± 2.1^‡^	12.8 ± 3.1
	Unemployed	10.7 ± 2.6	11.9 ± 2.2	11.3 ± 2.6^♦^	10.9 ± 2.3	10.1 ± 1.9^‡^	12.5 ± 3.3
HAART	Treated	11.2 ± 2.4	12.1 ± 2.4	11.5 ± 2.3	11.3 ± 2.5	10.8 ± 2.2	12.5 ± 3.1
	Naive	11.1 ± 2.3	11.9 ± 2.2	12.1 ± 2.7	11.1 ± 2.5	10.5 ± 2.1	12.6 ± 3.3

^†^ *P* = 0.021, *P = 0.024, ^♦^ *P* = 0.004, ^‡^ *P* = 0.001; rest of observed differences are not significant (*P* > 0.05).
